# Nitrous Oxide-induced B12 Deficiency Presenting With Myeloneuropathy

**DOI:** 10.7759/cureus.5331

**Published:** 2019-08-06

**Authors:** Ehizogie Edigin, Oyintayo Ajiboye, Avantika Nathani

**Affiliations:** 1 Internal Medicine, John H. Stroger Jr. Hospital of Cook County, Chicago, USA

**Keywords:** nitrous oxide, whippet, subacute combined degeneration of spinal cord, myeloneuropathy, vitamin b12 deficiency

## Abstract

Nitrous oxide abuse is an uncommon cause of subacute combined degeneration of the spinal cord. This is a case of a 27-year-old female exotic dancer who presented with clinical and laboratory manifestations of subacute combined degeneration of the spinal cord secondary to nitrous oxide-induced B12 deficiency from chronic whippets consumption.

## Introduction

Nitrous oxide is a drug of abuse among young adults in parties and night clubs [1]. It can be inhaled in the form of canisters. Nitric oxide interferes with the metabolism of B12 and can cause subacute combined degeneration of the spinal cord presenting with ataxia, paresthesia, loss of vibratory, and position sensation [2]. Symmetrical abnormally increased T2 signal intensity, confined to the posterior and posterior and lateral columns in the cervical and thoracic spinal cord, is the classic MRI finding [3]. B12 supplementation and cessation of nitrous oxide abuse is the mainstay of treatment and recovery can be slow and incomplete [4].

## Case presentation

A 27-year-old female with no significant past medical history presented with unsteadiness while walking, "pins and needles" sensation, and weakness in her lower extremities. Symptoms started about one week ago and have progressively worsened. Vital signs were within normal limits. On physical exam, she had impaired vibration, proprioception, and spasticity in both lower extremities. Pain and temperature sensation, reflexes, and cranial nerve testing were preserved. She had an ataxic gait and positive Romberg's test. Laboratory testing was remarkable for low B12 level of 105 nanograms/liter, hemoglobin of 10 g/dl, mean corpuscular volume of 105 femtoliters, homocysteine level of 20 micromoles/liter, and methylmalonic level of 0.9 micromoles per liter. Folate level, hemoglobin A1C, HIV, and syphilis testing were normal. On further interview, she endorsed inhaling on an average about 20-30 canisters of nitrous oxide in the form of whippets per day for the past one year. She works as an exotic dancer in a local night club and abuses nitrous oxide recreationally at work. For the past month, she has increased her consumption of nitric oxide. She endorsed having similar symptoms a couple of months ago. She self-medicated by injecting herself with B12 shots on advice from a friend, which led to the resolution of her symptoms.

She was diagnosed with nitrous oxide-induced B12 deficiency leading to myeloneuropathy. In the context of convincing clinical and laboratory features, further imaging was not pursued. The patient was started on intramuscular cyanocobalamin 1000 ug daily for five days and discharged on oral B12 supplementation. The patient was counseled to abstain from nitrous oxide abuse. She was seen in the neurology clinic after three months and had a near-complete resolution of symptoms. She had some residual paresthesia; however, symptoms were much improved compared to the initial presentation.

## Discussion

Nitrous oxide is a colorless, non-inflammable gas with a weak anesthetic but useful analgesic action [5]. Nitrous oxide misuse has gained popularity recently at parties and music festivals. The 2012 Global Drug Survey, an international online survey of drug use in young adults, reported that almost half of respondents had used nitrous oxide recreationally at some point, 10% within the preceding 12 months [1]. It is relatively cheap, available in night clubs in the form of whippets which can be inhaled for recreational purposes. Nitrous oxide interferes with vitamin B12 metabolism, by oxidizing the cobalt atom and irreversibly inactivating the enzyme methionine synthetase [6]. This impairs the production of methionine (from homocysteine), a substrate for tetrahydrofolate and thymidine during DNA synthesis.

**Figure 1:**
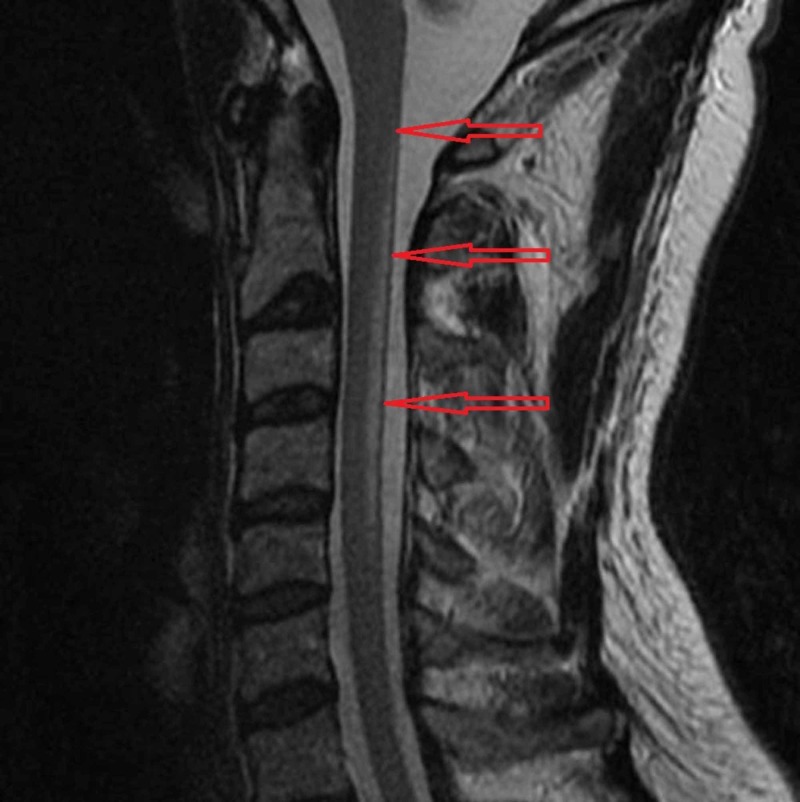
MRI reveals abnormal high T2 cervical cord signal within the dorsal columns extending from C2 to C5 highlighted by the red arrows The location of the signal abnormality is consistent with subacute combined degeneration of the cord. Image courtesy: [11]

The classic neurologic manifestations of cobalamin deficiency occur in the spinal cord, as subacute combined degeneration (SCD): impairment of the posterior columns causes loss of position and vibration sense, and damage to the corticospinal tracts results in weakness and spasticity [7]. Patients may present with sensory ataxia leading to a positive Romberg test, peripheral neuropathy leading to paresthesia, urinary retention, optic atrophy, and dementia [7-8]. A study of 143 patients with cobalamin deficiency demonstrated that the most common findings were combined myelopathy and neuropathy (41% of patients), followed by isolated neuropathy (25%) or myelopathy (12%), and cognitive impairment (8%) [9]. The spinal cord involvement is associated with the most frequent clinical manifestation of vitamin B12 deficiency, namely SCD. The most consistent MRI finding in SCD is symmetrical abnormally increased T2 signal intensity (Figure 1), commonly confined to posterior or posterior and lateral columns in the cervical and thoracic spinal cord [3]. MRI is not needed for diagnosis in the right clinical and laboratory setting. In most cases of nitrous oxide-induced neurological dysfunction, the serum B12 concentration is low in the presence of macrocytic anemia. B12 levels can, however, be normal. In these cases of functional B12 deficiency, serum levels of homocysteine, and methylmalonic acid will be elevated. Methylmalonic acid is more specific for B12 deficiency as folate deficiency can also cause elevated homocysteine levels [10].

Due to the wide variety of neurologic, hematologic, and radiologic findings associated with cobalamin deficiency, it is important to rule out conditions that may have a similar presentation. Primary folic acid, mercury toxicity, copper deficiency, Guillain-Barré syndrome, and multiple sclerosis can share some similarities with B12 deficiency [7]. A common approach to treatment is intramuscular cyanocobalamin 1000 μg daily for a minimum of five days, followed by continued intermittent dosing until symptoms abate. The literature demonstrates that with cyanocobalamin supplementation and abstinence from nitrous oxide use, significant-to-total recovery from SCD can occur within 14 days to 21 months [4]. An observational study reviewed outcomes in 57 cases of SCD and showed that although 86% of patients treated with cyanocobalamin had some recovery from neurologic symptoms, only 14% had total recovery at a range of 1-84 weeks after diagnosis [12]. Hence, recovery from nitrous oxide neuropathy may be slow and incomplete, despite high-dose vitamin B12 replacement.

## Conclusions

Nitrous oxide abuse can lead to subacute combined degeneration of the spinal cord due to interferences with B12 metabolism. A patient may present with low or normal B12 levels in the presence of elevated homocysteine and methylmalonic levels. Physicians should suspect possible nitrous oxide abuse in young patients with neurologic manifestations of B12 deficiency without any other alternative etiologies.
